# Reply to Mitsuboshi, “Enhancing result reliability by addressing potential confounding factors”

**DOI:** 10.1128/spectrum.01499-24

**Published:** 2025-02-06

**Authors:** Tomoyuki Ishigo, Kazuaki Matsumoto, Hiroaki Yoshida, Hiroaki Tanaka, Yuta Ibe, Satoshi Fujii, Masahide Fukudo, Hisato Fujihara, Fumihiro Yamaguchi, Fumiya Ebihara, Takumi Maruyama, Yukihiro Hamada, Masaru Samura, Fumio Nagumo, Toshiaki Komatsu, Atsushi Tomizawa, Akitoshi Takuma, Hiroaki Chiba, Yoshifumi Nishi, Yuki Enoki, Kazuaki Taguchi, Ayako Suzuki

**Affiliations:** 1 Department of Pharmacy, Sapporo Medical University Hospital, Sapporo, Japan; 2 Division of Pharmacodynamics, Faculty of Pharmacy, Keio University, Tokyo, Japan; 3 Department of Pharmacy, Kyorin University Hospital, Mitaka, Japan; 4 Department of Pharmacy, Showa University Fujigaoka Hospital, Yokohama, Japan; 5 Department of Hospital Pharmaceutics, School of Pharmacy, Showa University, Tokyo, Japan; 6 Department of Respiratory Medicine, Showa University Fujigaoka Hospital, Yokohama, Japan; 7 Department of Pharmacy, Tokyo Women’s Medical University Hospital, Tokyo, Japan; 8 Department of Pharmacy, Kochi Medical School Hospital, Kochi, Japan; 9 Department of Pharmacy, Yokohama General Hospital, Yokohama, Japan; 10 Department of Pharmacy, Kitasato University Hospital, Sagamihara, Japan; 11 Department of Pharmacy, Showa University Northern Yokohama Hospital, Yokohama, Japan; 12 Department of Pharmacy, Tohoku Kosai Hospital, Sendai, Japan; 13 Center for Pharmacist Education, School of Pharmacy, Nihon University, Funabashi, Japan; University of Pretoria, Pretoria, Gauteng, South Africa

**Keywords:** vancomycin, therapeutic drug monitoring, nephrotoxicity, area under the concentration–time curve

## REPLY

We thank Dr. Mitsuboshi for his comments (1) on our recent article, “Relationship Between Nephrotoxicity and Area Under the Concentration-Time Curve of Vancomycin in Critically Ill Patients: a Multicenter Retrospective Study” (2).

The Cox proportional hazards model is acknowledged to have limitations because it fails to account for mortality risk. Therefore, we estimated hazard ratios (HRs) for AKI using a Fine–Gray model adjusted for mortality as a competing risk. Patients with an AUC on day 2 (AUC_24–48 h_) of <500 µg·h/mL were the reference controls for comparison with different AUC_24–48 h_ groups. In the Fine–Gray model, we adjusted for an creatinine-based estimated glomerular filtration rate (eGFRcre) of <30 mL/min/1.73 m², the use of tazobactam/piperacillin (TZP), and the use of loop diuretics, as these factors are associated with an increased risk of AKI. The intermediate-area under the concentration–time curve (AUC) group (HR, 5.4 [95% confidence interval {CI}, 1.75–16.46]; *P* = 0.003), high-AUC group (HR, 7.0 [95% CI, 2.39–20.61]; *P* < 0.001), and the use of TZP (HR, 3.2 [95% CI, 1.15–8.83]; *P* = 0.026) were significantly associated with a higher incidence of AKI (Table 1).

**TABLE 1:** Fine–Gray analyses of factors associated with AKI^*a*^*^,^*^*b*^

	Univariate model			Multivariate model A		
	HR	95% CI	*P* value	HR	95% CI	*P* value
Age, per 1 year increase	1.0	0.98–1.04	0.380			
Sex: female	0.8	0.34–1.92	0.630			
BMI, per 1 kg/m^2^ increase	1.0	0.94–1.11	0.690			
SOFA score	1.0	0.92–1.16	0.580			
APACHE II score	1.0	0.91–1.11	0.910			
Sepsis	1.0	0.42–2.36	1.000			
Septic shock	1.8	0.74–4.28	0.200			
VAN AUC_24–48 h_						
<500 µg·h/mL	Reference	Reference	Reference	Reference	Reference	Reference
500–600 µg·h/mL	3.7	1.38–9.82	0.009	5.4	1.75–16.46	0.003
≥600 µg·h/mL	6.6	2.30–18.93	<0.001	7.0	2.39–20.61	<0.001
Loding dose, ≥25 mg/kg	1.0	0.40–2.25	0.910			
Maintenance dose (mg/kg/day)	1.0	0.93–1.07	0.880			
eGFRcre, <30 mL/min/1.73 m^2^	0.6	0.08–4.45	0.611	1.0	0.09–9.75	0.970
BUN:Scr, ≥20	1.1	0.39–3.40	0.810			
TZP	2.1	0.80–5.42	0.130	3.2	1.15–8.83	0.026
Catecholamine	2.1	0.85–5.07	0.110			
Loop diuretic	1.2	0.49–2.88	0.700	0.8	0.34–1.86	0.600

^*a*^ Statistical significance was set at *P* <0.05. To account for the increased alpha error, the Bonferroni correction was applied to compare the three groups. *P* values less than 0.0167 were considered significant.

^*b*^ Abbreviations: AKI, acute kidney injury; APACHE II, Acute Physiology and Chronic Health Evaluation II; AUC, area under the concentration–time curve; AUC_24–48 h_, AUC on day 2; BMI, body mass index; BUN:Scr, ratio of blood urea nitrogen to serum creatinine; CI, confidence interval; eGFRcre, creatinine-based estimated glomerular filtration rate; HR, hazard ratio; SOFA, sequential organ failure assessment; TZP, tazobactam/piperacillin; VAN, vancomycin.

We calculated a doubly robust (DR) estimator for the incidence of AKI that accounts for the inverse probability of treatment weighting (IPTW) at baseline and compared the average treatment effects (ATEs) with other estimators. In addition, we examined the association between each AUC_24–48 h_ and the occurrence of AKI after adjustment by the propensity score (PS) or IPTW. We used PS or IPTW adjustments to balance the risk factors for AKI in the low- and moderate- to high-AUC groups. The PS for the probability of selecting a low-AUC group was calculated based on patient characteristics at baseline, including age, sex, body mass index, sepsis, sequential organ failure assessment (SOFA) score, Acute Physiology and Chronic Health Evaluation II (APACHE II) score, ratio of blood urea nitrogen to serum creatinine, albumin levels, eGFRcre <30 mL/min/1.73 m², usage of TZP, aminoglycosides, loop diuretics, catecholamines, angiotensin-converting enzyme inhibitors/angiotensin II receptor blocker, and nonsteroidal anti-inflammatory drugs. Imputation of missing data for SOFA and APACHE II scores was performed using a multivariate normal distribution. The IPTW was calculated using the PS. AKI risk analysis resulted in a DR estimator of 0.1503 and an ATE of 0.3653 (0.0527 for the low-AUC group and 0.4180 for the intermediate- to high-AUC group). Similarly, the estimator when adjusted for PS was 0.1323, and ATE was 0.2515 (0.0645 and 0.3160), and the estimator when adjusted for IPTW was 0.1252, and ATE was 0.2457 (0.0596 and 0.3053). The actual incidence of AKI was 14% and the ATE was 26.8% (6.5% for the low-AUC group and 33.3% for the intermediate- to high-AUC group), indicating robustness. Furthermore, compared with the low-AUC group, the intermediate-AUC group and high-AUC group showed a significantly higher risk of AKI (Table 2).

**TABLE 2:** Logistic analyses of factors associated with AKI^*a*^*^,^*^*b*^

	Model B			Model C		
	OR	95% CI	*P* value	OR	95% CI	*P* value
VAN AUC_24–48 h_						
<500 µg·h/mL	Reference	Reference	Reference	Reference	Reference	Reference
500–600 µg·h/mL	5.8	1.48–22.70	0.012	5.0	1.39–18.26	0.014
≥600 µg·h/mL	12.8	3.03–54.17	<0.001	11.3	2.77–46.52	<0.001

^*a*^ Statistical significance was set at *P* <0.05. To account for the increased alpha error, the Bonferroni correction was applied to compare the three groups. *P* values less than 0.0167 were considered significant. Model B was adjusted by PS score, and model C was adjusted by IPTW.

^*b*^ Abbreviations: AKI, acute kidney injury; AUC, area under the concentration-time curve; AUC_24–48 h_, AUC on day 2; eGFRcre, creatinine-based estimated glomerular filtration rate; CI, confidence interval; IPTW, inverse probability of treatment weighting; OR, odds ratio; PS, propensity score; VAN, vancomycin.

Together, our findings demonstrated the correlation between the AUC on day 2 of vancomycin and the incidence risk of AKI in intensive care unit patients, suggesting that AUCs between 500 and 600 µg·h/mL as well as those above 600 µg·h/mL are also associated with an increased risk of AKI. We believe that this robust additional analysis will improve the reliability of this study.
